# Supplementary material for: Flexible Coatings Facilitate pH-Targeted Drug Release via Self-Unfolding Foils: Applications for Oral Drug Delivery
Source: Pharmaceutics. 2024 Jan 6;16(1):81. doi: 10.3390/pharmaceutics16010081 (PMC10819044; doi:10.3390/pharmaceutics16010081)
Supplement: Supplementary file 1 [file pharmaceutics-16-00081-s001.zip › pharmaceutics-2783831-supplementary.pdf]

Supplementary Material

Flexible Coatings Facilitate pH-Targeted Drug Release via Self-Unfolding Foils: Applications for Oral Drug Delivery

Carmen Milián-Guimerá *,†, Laura De Vittorio *,†, Reece McCabe, Nuray Göncü, Samvrta Krishnan, Lasse Højlund Eklund Thamdrup, Anja Boisen and Mahdi Ghavami

The Danish National Research Foundation and Villum Foundation's Center for Intelligent Drug Delivery and Sensing Using Microcontainers and Nanomechanics, Department of Health Technology, Technical University of Denmark, 2800 Kgs. Lyngby, Denmark

* Correspondence: camigu@dtu.dk (C.M.-G.); ladevi@dtu.dk (L.D.V.)

† These authors contributed equally to this work.

Table S1. Thickness measurements of the different polymeric mixtures.

Table S1. Thickness of the respective enteric polymeric mixtures (mean ± SD, n = 3).

	Thickness (µm)
**EFL**	26.63 ± 0.77
**E0**	26.98 ± 1.99
**E12.5**	29.21 ± 0.57
**E25**	27.79 ± 1.27
**K0**	21.49 ± 0.83
**K12.5**	23.44 ± 0.34
**K25**	24.71 ± 0.41

Table S1 shows the individual thickness of the respective enteric polymeric mixtures used as a top coating during SUF preparation. The thickness was measured by contact profilometry (Alpha-Step IQ Stylus Profilometer, KLA-Tencor Corporation, Milpitas, USA). For that purpose, the different mixtures of the polymers were sprayed on flat silicon chips using the SUF spray coating protocol outlined above. The coating thicknesses were measured by making a scratch in the coating and running measurements across it using a 3 mg tip force with a scan speed of 130 µm/s and a resolution of 0.4 µm. All measurements were performed with three replicates for each polymer mixture and repeated a total of three times in different locations of each sample. Visualization of the loaded and coated foils, before and after fitting into a capsule, was carried out by SEM.

S1. SEM image of a cross-section of an empty SUF.

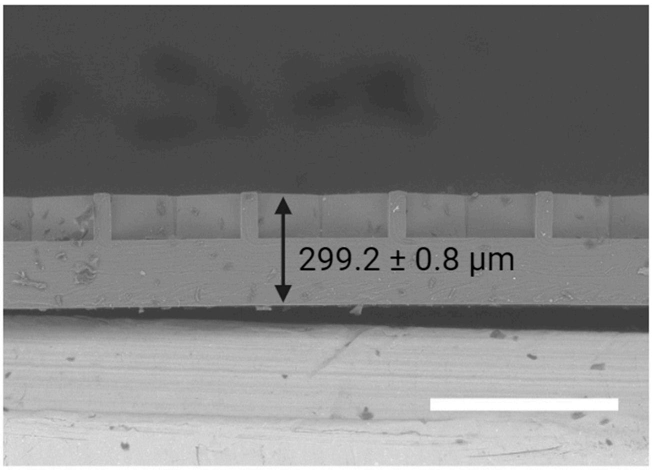

Figure S1. SEM image of a cross-section of an empty SUF and its respective thickness (mean ± SD, n = 4). The scale bar represents 500 µm.

S2. Individual stress-strain curves of tensile tests.

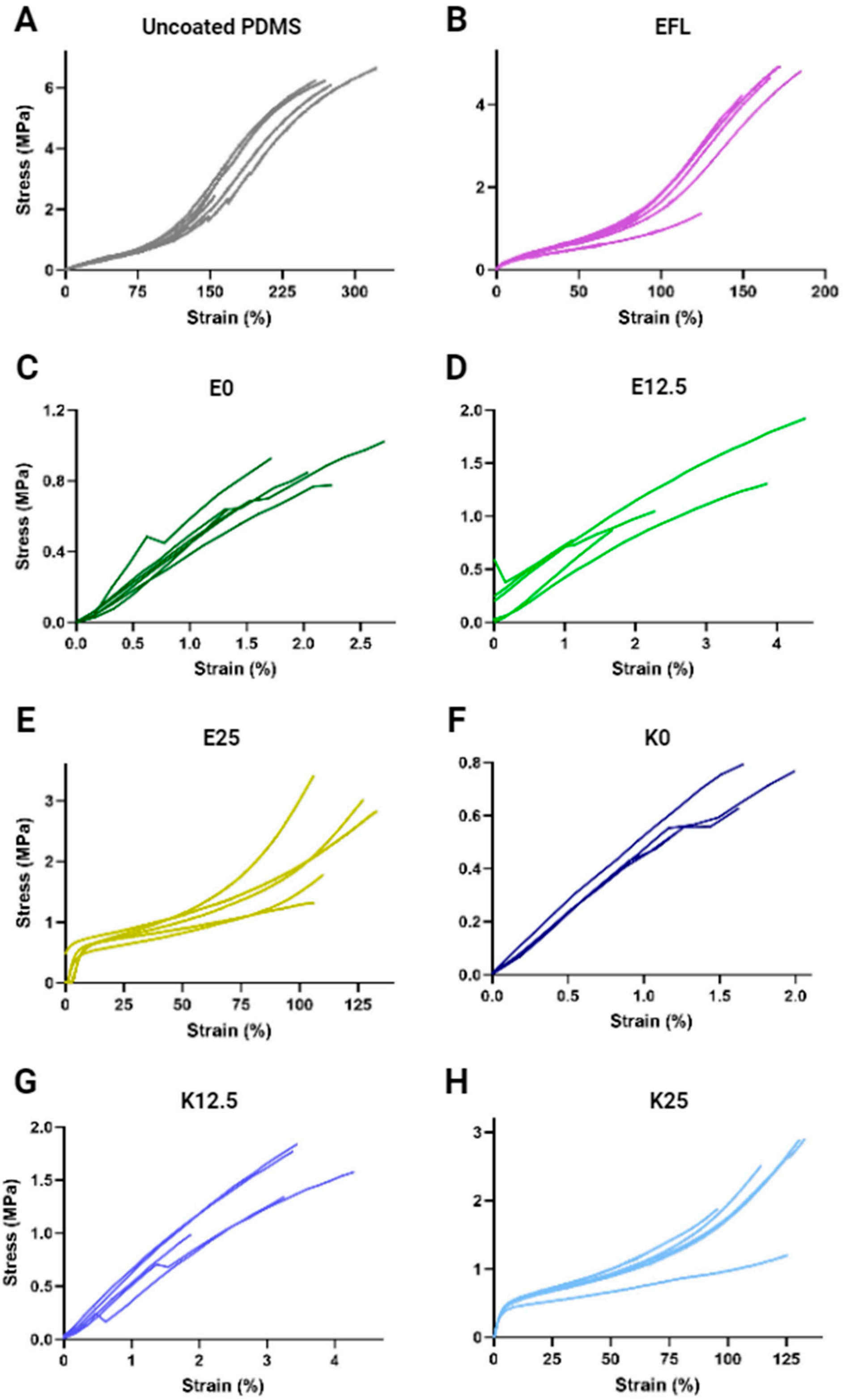

Figure S2. Individual stress-strain curves of (**A**) uncoated PDMS as a reference and PDMS spray coated with (**B**) EFL, (**C**) E0, (**D**) E12.5, (**E**) E25, (**F**) K0, (**G**) K12.5 and (**H**) E25 polymeric mixtures. The measurements were performed within a total of n = 4-5 replicates.

Figure S2 shows the individual stress-strain curves of the enteric mixtures spray coated onto surface treated PDMS dogbones. The measurements were performed by using a Texture Analyzer (TA.XTplusC Texture Analyzer, Stable Micro Systems, Godalming, England). A 10 kg load cell and a A/TG screw-initiated vice clamp operating on knurled jaw faces (35 mm × 35 mm) was used to carry out the pull test at a constant rate of 1 mm/s. Each type of sample was measured with 4-5 replicates.
